# Supplementary material for: Patient satisfaction in regional referral hospitals of Bhutan: Insights from a cross-sectional study
Source: PLoS One. 2025 Jun 2;20(6):e0312629. doi: 10.1371/journal.pone.0312629 (PMC12129210; doi:10.1371/journal.pone.0312629)
Supplement: Attachment — Submitted filename: Reviewers comments.docx [file pone.0312629.s001.docx]

Abstract

1. Rather than saying "archived patient satisfaction data," you could clarify the source or specific nature of this data.

Introduction

The introduction provides a solid contextual foundation for the study. Minor stylistic adjustments would improve readability and conciseness, particularly by condensing and refining some sentences.

1. The phrase “meeting patient’s needs is imperative” could be made more specific, and the sentence starting “Starting from the early 1980s” might benefit from a more concise phrasing.
2. Also, the lack of a universally accepted definition of patient satisfaction could be condensed to focus more on its role as a quality measure.
3. The phrase “meticulously developed tool” to describe the PSQ-18 may appear overly emphatic in a scientific context.

Materials and methods

1. Confirm whether the survey sites were called “MERRH” or “ERRH” and “GCRRH” or “CRRH”.
2. Consider adding a brief statement on the justification for modifying specific terminology in the PSQ-18 and whether there was any assessment of the adapted tool’s reliability or validity in this new context.
3. Add a sentence specifying the handling of negative items during re-scaling, as this will aid replicability and ensure clarity in interpretation.
4. Briefly explain what the Dunnett grouping is, why the Dunnett grouping was used to identify distinct groups and why a significance level of 0.005 was selected. The significance level of 0.005 is strict and unusual (more commonly 0.05), so a rationale for this choice would also improve clarity.

Result

1. Satisfaction scores vary significantly by age, with older respondents generally reporting higher satisfaction levels. This finding could be influenced by generational differences in expectations of healthcare, which might warrant a brief discussion of the interpretation.
2. The study shows a predominant representation of Sharchop ethnic group respondents (57.05%), and satisfaction scores are also the highest among them. Given Bhutan's ethnic diversity, exploring whether this reflects the general population distribution or biases in sampling might be helpful.
3. Illiterate individuals reported higher satisfaction scores than those with higher education levels, which might suggest varying expectations based on education. This could be discussed as it may reveal important insights into patient perceptions of healthcare quality across different educational backgrounds.
4. The choice of control groups (e.g., using the 18-44 age group or married status as the control) appears sound. However, mentioning why these groups were selected as the baseline for comparison could strengthen interpretability.
5. Given that significant differences in satisfaction levels were observed between MERRH and GCRRH, it may be beneficial to explore specific differences in facilities, staff interaction, or procedural efficiency between the two. These insights could further contextualize why one center reports higher satisfaction levels.

Discussion

1. The authors astutely highlight Buddhism's influence on Bhutanese healthcare interactions, leading to positive patient responses. However, there may be potential biases, as indicated, that result from cultural expectations of politeness and contentment, particularly when surveys are administered by quality units. It may be valuable to propose alternative assessment methods, such as third-party evaluators or anonymous surveys, to better gauge satisfaction independently of cultural expectations.
2. The potential for satisfaction surveys to affect provider performance and motivation is a valid concern. The authors might expand this discussion by considering how Bhutan could develop a model where satisfaction surveys are used solely for improvement and not linked to individual compensation or evaluations, thus mitigating potential professional dissatisfaction and unintended care implications.

Conclusion

1. The authors could suggest areas for further research, such as longitudinal studies or comparative studies with similar healthcare systems in culturally similar countries. This would aid policymakers in anticipating and addressing shifts in patient expectations and satisfaction.

Recommendations

1. If the paper could discuss the limitations explicitly if any would strengthen the conclusions.
2. Qualitative follow-up with respondents could add depth to the quantitative findings, helping to contextualize why specific groups, such as the illiterate population, report higher satisfaction.
