# Supplementary material for: Patient satisfaction in regional referral hospitals of Bhutan: Insights from a cross-sectional study
Source: PLoS One. 2025 Jun 2;20(6):e0312629. doi: 10.1371/journal.pone.0312629 (PMC12129210; doi:10.1371/journal.pone.0312629)
Supplement: Attachment — Submitted filename: Response to Reviewers.docx [file pone.0312629.s004.docx]

**Response to Reviewers**

**Reviewer #1**

**Study title:**
**Comment:** You need to re-write the study title as follows
“Evaluation of patient satisfaction in the regional referral hospitals in Bhutan: a cross-sectional study”.

**Response:** Thank you for the comment. We have made the changes as follow:

“Patient satisfaction in regional referral hospitals of Bhutan: Insights from a Cross-Sectional Study”

**Abstract:**
**Comment:** You need to re-write the abstract in a standard format including the headings ‘Background, Methods, Results, and Conclusion’.

**Response:** Thank you for your valuable feedback. Initially, we provided the abstract in an unstructured format, consistent with the published articles in PLOS ONE, where abstract formatting varies. However, in light of your comment, we have revised the abstract into a structured format, aligning it with the conventions of structured abstracts for clarity and comprehensiveness.


**Main text:**
**Introduction:**
**Comment:** It is well-written and appreciated.

**Response:** Not required
 **Materials and methods:**
**Comment:** You need to re-write methods section including the following sub-headings as a separate entity:
-study design
-study site
-study duration
-study setting
-study population
-inclusion criteria
-exclusion criteria
-sample size determination
-sampling method
-study variables
-study procedures
-data management
-data analysis

**Response:** Dividing the Materials and Methods section into individual sub-headings as recommended would result in a very brief and fragmented write-up, which might not effectively present the detailed methodology of the study. To ensure a more cohesive and comprehensive narrative, we have grouped related sub-headings together into four broader categories, each addressing a key aspect of the study. This approach allows us to present the methodology concisely while maintaining the clarity and flow necessary for readers to understand the process.

The grouped sub-headings are as follows:

1. Study design and setting
2. Study population and survey instrument
3. Data management and analysis
4. Ethical considerations

**Results:**
**Comment:** In Table 1, you can compare the socio-demographic variables and clinical profiles of the respondents between Gelephu and Mongar Hospital, and that will give more meaning to the table.

**Response:** We sincerely thank the reviewer for their insightful comment. We agree that presenting the socio-demographic variables and clinical profiles of the respondents from both hospitals provides valuable additional insights. Accordingly, we have amended Table 1 to include these details, enabling a more meaningful comparison.

**Comment:** You have inappropriately used one-way ANOVA analysis. ANOVA can be used only if you have more than 2 groups to compare quantitative variables. So, you can delete current table 2, and construct new table 2 to compare patient satisfaction scores across seven domains between Gelephu and Mongar hospital.

**Response:** We sincerely thank the reviewer for their observation regarding the application of one-way ANOVA. We would like to clarify that while ANOVA is commonly used to compare more than two groups, it is also valid for comparing two groups. In fact, when there are exactly two groups, the results of a one-way ANOVA are mathematically equivalent to those obtained from a t-test. In our study, we applied one-way ANOVA consistently across all variables for uniformity, as some variables involve more than two groups (e.g., Age, Ethnic Group, and Marital Status).

To adhere to general practice, we have now used the 2-sample t-test for variables such as Age, Sex, and Service Utilisation, as these have only two groups. To clarify the statistical methods used, a footer has been added to the table specifying where the one-way ANOVA and t-test have been performed. However, there have been no changes in the significance level.

We would like to express our sincere thanks for your insightful suggestion to compare patient satisfaction across the seven domains between the two hospitals. We agree that comparing patient satisfaction across the seven domains between the two hospitals is a valid approach to analyzing the data. However, as we have already provided an overall comparison of patient satisfaction between Gelephu and Mongar hospitals in table 2, we believe that focusing on patients' socio-demographic and clinical variables offers more meaningful insights for actionable improvements at a national level. Both hospitals are state-owned, with comparable services and infrastructure, making them suitable for broader comparisons.

Rather than comparing hospital-to-hospital satisfaction across the domains, we chose to examine factors such as age, education, and occupation, as these are critical in identifying specific groups that may benefit from targeted interventions. This approach aligns with the goal of improving patient satisfaction more effectively across the country, addressing the needs of diverse population groups, and guiding healthcare policies.

While we understand and acknowledge the value of direct hospital comparisons, we firmly believe that examining the socio-demographic and clinical variables will provide a more comprehensive understanding of the factors driving patient satisfaction, with direct implications for policy and practice at a national level.

**Comment:** You need to re-write table 3 to include univariate analysis in one column and multivariate logistic regression analysis in another column. This will give a better understanding to the reader about the factors associated with poor patient satisfaction or well-satisfied patient.

**Response:** We sincerely thank the reviewer for their insightful feedback and suggestion to include univariate analysis alongside multivariate logistic regression analysis. Patient satisfaction is a complex outcome influenced by multiple interconnected factors. Univariate analysis, while useful for initial explorations, might provide misleading information by failing to account for confounding variables. For example, a variable may appear to have a significant association with patient satisfaction in univariate analysis but lose its significance when adjusted for other factors. Conversely, some associations may only emerge after adjusting for confounders.

Given the multifactorial nature of patient satisfaction, we believe that presenting adjusted odds ratios derived from multivariate logistic regression provides a more accurate and meaningful understanding of the factors associated with satisfaction. To enhance clarity, we have amended the table to explicitly label the odds ratios as "Adjusted Odds Ratios"

**Comment:** Table 4 doesn’t give any scientific inferences. You can delete table 4.

**Response**: Thank you for your feedback. Table 4 provides a detailed breakdown of satisfaction levels across different socio-demographic groups and domains of care. This information is crucial for identifying specific areas that require improvement. In our experience, such descriptive details are essential for understanding the nuances of patient satisfaction, especially when striving to enhance the quality and safety of healthcare service delivery. By presenting these details, we aim to offer insights that can guide targeted interventions, ensuring that improvements are aligned with the needs of different patient groups. We believe that these specifics are key to informed decision-making and evidence-based policy formulation.

**Reviewer #2**

**Abstract**
**Comment:** Rather than saying "archived patient satisfaction data," you could clarify the source or specific nature of this data.

**Response:** To address the reviewer's comment, the section in the abstract has been revised as follows:

“We conducted a retrospective analysis of patient satisfaction survey responses archived in the quality assurance unit of two tertiary healthcare centres in Bhutan: Mongar Eastern Regional Referral Hospital and Gelephu Central Regional Referral Hospital. The routine surveys, administered throughout April 2024, utilised an adapted version of the Patient Satisfaction Questionnaire-18. The data were analysed using descriptive and inferential statistics.”

**Introduction**
The introduction provides a solid contextual foundation for the study. Minor stylistic adjustments would improve readability and conciseness, particularly by condensing and refining some sentences.
**Comment:** The phrase “meeting patient’s needs is imperative” could be made more specific, and the sentence starting “Starting from the early 1980s” might benefit from a more concise phrasing.

**Response:** Thank you for the suggestion. We chose to keep the phrase "meeting patients' needs is imperative" broad because patient needs are inherently diverse and encompass various dimensions (e.g., clinical, emotional, psychological, etc.). Listing all potential needs would risk being overly exhaustive and detract from the statement's clarity.

Regarding the sentence starting with “Starting from the early 1980s,” we agree that conciseness can enhance readability. The revised section now reads:

“Since the early 1980s, there have been ongoing efforts to comprehend and measure patient satisfaction, and gradually, it has been acknowledged as a crucial component in delivering quality healthcare.”

**Comment:** Also, the lack of a universally accepted definition of patient satisfaction could be condensed to focus more on its role as a quality measure.

**Response:** Throughout the document, we have emphasized the role of patient satisfaction as a measure of quality. While we agree that the focus should remain on its positive impact, we also believe it is essential to acknowledge its inherent ambiguity. Therefore, we felt it necessary to mention the lack of a universally accepted definition, which reflects this ambiguity. Additionally, the sentence in question is brief, consisting of only 14 words, and we believe it does not detract from the overall clarity of the manuscript.

**Comment:** The phrase “meticulously developed tool” to describe the PSQ-18 may appear overly emphatic in a scientific context.

**Response:** Thank you for the feedback. We agree that the term 'meticulously developed tool' might be overly emphatic in a scientific context. We have revised the sentence to ensure a more neutral tone:

“To gauge patient satisfaction, Bhutanese healthcare centres utilize the Patient Satisfaction Questionnaire 18 (PSQ-18), a well-validated tool noted for its brevity and efficacy across various contexts.”

**Materials and methods**
**Comment:** Confirm whether the survey sites were called “MERRH” or “ERRH” and “GCRRH” or “CRRH”.

**Response:** Thank you for the feedback. Currently, at the ministry level, there is no consensus on the official terminology, except for the use of "regional referral hospital." Both in official and non-official correspondences, abbreviations such as MERRH/ERRH/MRRH and GCRRH/CRRH/GRRH have been used interchangeably.

According to the official websites of the hospitals, Mongar is referred to as the Mongar Regional Referral Hospital (https://www.mrrh.gov.bt/), while Gelephu is called the Central Regional Referral Hospital (http://www.sarpang.gov.bt/institution/central-regional-referral-hospital), further highlighting this inconsistency. Moreover, an enquiry with the hospital administration confirmed the lack of a standardized term and no official confirmation from the ministry. In light of this, we believe there is no need to change the abbreviations of the study sites at this time.

**Comment:** Consider adding a brief statement on the justification for modifying specific terminology in the PSQ-18 and whether there was any assessment of the adapted tool’s reliability or validity in this new context.

**Response:** Thank you for the feedback. The modifications made to the PSQ-18 in the hospital’s survey are detailed in the Methodology section. There were no major changes to the original questionnaire; the only adjustments were the replacement of the terms "doctor" and "doctor’s office" with "healthcare professionals" and "healthcare centre," respectively. These modifications were made to ensure the tool's broader applicability across various healthcare settings while preserving the original intent of the questions. To prevent any confusion, we have replaced the term "modified version" with "adapted version," as the changes did not alter the original structure or purpose of the tool.

As this is a retrospective study, there is limited scope for conducting a formal assessment of the tool's reliability or validity in this new context. Given that the adaptations did not impact the core framework of the PSQ-18, no additional reliability or validity testing was performed.

**Comment:** Add a sentence specifying the handling of negative items during re-scaling, as this will aid replicability and ensure clarity in interpretation.

**Response:** Thank you for your comment. The handling of negative items during re-scaling has been outlined in the Methodology section. We followed the standard re-scaling procedure for the PSQ-18 negative items, as recommended in the published guidelines, and applied it consistently in our analysis. Since this is a well-established practice, we did not feel it necessary to provide an exhaustive description in the methodology. For further reference, the published article detailing this procedure can be accessed via the following link: <https://www.rand.org/content/dam/rand/www/external/health/surveys_tools/psq/psq18_scoring.pdf>

**Comment:** Briefly explain what the Dunnett grouping is, why the Dunnett grouping was used to identify distinct groups and why a significance level of 0.005 was selected. The significance level of 0.005 is strict and unusual (more commonly 0.05), so a rationale for this choice would also improve clarity.

**Response:** Performing one-way ANOVA on Minitab provides the following grouping information:

1. Tukey
2. Fisher
3. Dunnett
4. Hsu MCB
5. Games-Howell

Although all the aforementioned grouping tests were performed, only the results of Dunnett's comparison were reported in the manuscript. The findings from all methods were consistent, with no significant differences observed. It is generally recommended to focus on specific group comparisons rather than conducting an overall comparison, if possible, as these methods tend to be more powerful. For example, Tukey's method generally provides less precise confidence intervals and less powerful hypothesis tests compared to Dunnett's or Hsu’s MCB.

In our study, since we already have a mean satisfaction level for each subgroup, we opted to use either the lowest or highest score as a reference point. This approach allowed for more precise and powerful comparisons, aligning to enhance statistical accuracy in our analysis.

Thank you for noting the significance level. Due to a typographical error, we initially mentioned a significance level of 0.005; however, all analyses were conducted at a significance level of 0.05. The correction has been applied.

**Result**
**Comment:** Satisfaction scores vary significantly by age, with older respondents generally reporting higher satisfaction levels. This finding could be influenced by generational differences in expectations of healthcare, which might warrant a brief discussion of the interpretation.

**Response:** In the Results section, we have presented only the findings, in accordance with standard practice. Brief discussions of these findings are provided in the Discussion section, specifically between lines 229 and 235. Additionally, in lines 213 to 215, we have discussed the rising literacy rate and its potential negative impact on patient satisfaction. The generational difference in healthcare expectations can be significantly influenced by literacy levels, and this has been addressed accordingly.

**Comment:** The study shows a predominant representation of Sharchop ethnic group respondents (57.05%), and satisfaction scores are also the highest among them. Given Bhutan's ethnic diversity, exploring whether this reflects the general population distribution or biases in sampling might be helpful.

**Response:** Thank you for your comment. As noted in the published literature (e.g., The Kingdom of Bhutan Health System Review), there is no available data on the precise population size of each ethnic group in Bhutan. However, it is our assumption that the Sharchop represent a significant portion of the population, which is reflected in their higher representation in this study. Given that the Eastern Regional Referral Hospital (MERRH) is located in the eastern region, where the Sharchop form the majority, their dominance in the study population is consistent with the regional demographic.

The original survey involved collecting feedback from every fifth patient (mentioned in the methodology), minimizing any potential sampling bias from the outset. Furthermore, all archived patient satisfaction data with complete details were included in the study to ensure no bias in data selection.

Regarding the underrepresentation of the Ngalop population, we have acknowledged this limitation in the Discussion section of the manuscript.

**Comment:** Illiterate individuals reported higher satisfaction scores than those with higher education levels, which might suggest varying expectations based on education. This could be discussed as it may reveal important insights into patient perceptions of healthcare quality across different educational backgrounds.

**Response:** In the Results section, we have presented only the findings, in accordance with standard practice. Brief discussions of these findings are provided in the Discussion section, specifically between lines 206 and 212.

**Comment:** The choice of control groups (e.g., using the 18-44 age group or married status as the control) appears sound. However, mentioning why these groups were selected as the baseline for comparison could strengthen interpretability.

**Response:** Thank you for your comment. We agree that explaining the selection of control groups would enhance the clarity of our methodology. In our analysis, the reference group was selected based on its ability to provide clear and meaningful comparisons. For some analyses, the highest satisfaction score was used as the reference, and for others, the lowest satisfaction score was used. This dynamic approach allowed us to capture the nuances of group differences more effectively.

We have now amended the methodology section to provide additional details on the Dunnett grouping. The revised methodology reads as follows:

“Subsequently, Dunnett’s test was applied to identify statistically distinct groups within these predefined categories. Depending on which provided the most interpretable results, the group with the lowest or the highest mean score was used as the comparison reference.”

**Comment:** Given that significant differences in satisfaction levels were observed between MERRH and GCRRH, it may be beneficial to explore specific differences in facilities, staff interaction, or procedural efficiency between the two. These insights could further contextualize why one center reports higher satisfaction levels.

**Response:** In the Results section, we have presented only the findings, in line with standard practice. A brief discussion of these findings is provided in the Discussion section, specifically between lines 259 and 264, as follows:

“The MERRH and GCRRH are both state-owned healthcare centres with comparable infrastructure and resources; nevertheless, patient satisfaction is significantly higher at MERRH. This difference might be attributed to the socio-demographic characteristics of the population served. MERRH, located in eastern Bhutan, predominantly serves Sharchop individuals, who are significantly more satisfied than the Lhotshampa individuals primarily served by GCRRH in south-central Bhutan.”

Given the existing data, we can only suggest that ethnic differences between the populations served may account for the observed satisfaction levels. Further research is needed to explore specific factors, such as differences in facilities, staff interaction, or procedural efficiency, that may contribute to this variation. Our findings warrant additional studies to better understand the underlying factors responsible for the differences observed.

**Discussion**
**Comment:** The authors astutely highlight Buddhism's influence on Bhutanese healthcare interactions, leading to positive patient responses. However, there may be potential biases, as indicated, that result from cultural expectations of politeness and contentment, particularly when surveys are administered by quality units. It may be valuable to propose alternative assessment methods, such as third-party evaluators or anonymous surveys, to better gauge satisfaction independently of cultural expectations.

**Response:** We thank the reviewers for their valuable feedback. In our previous manuscript, we had only mentioned the need to promote awareness regarding the importance of feedback. We have now revised this section, and it reads as follows:

“Our study shows high overall satisfaction among Bhutanese patients across all seven domains. This finding highlights the concerted efforts of the Bhutanese healthcare system to ensure the provision of quality healthcare services through a comprehensive and systematic approach, including the vigorous implementation of the BHSQA. However, it is crucial to acknowledge that the surveys are administered by the quality unit of respective healthcare centres, potentially introducing a bias toward positive responses. To mitigate this bias, healthcare centres could implement online and anonymous survey systems or engage external evaluators to assess patient satisfaction”

**Comment:** The potential for satisfaction surveys to affect provider performance and motivation is a valid concern. The authors might expand this discussion by considering how Bhutan could develop a model where satisfaction surveys are used solely for improvement and not linked to individual compensation or evaluations, thus mitigating potential professional dissatisfaction and unintended care implications.

**Response:** We appreciate the reviewer’s insightful comment and have amended the document as recommended. The revised section now reads as follows:

“In Bhutan, where the healthcare quality culture is still evolving, utilising patient satisfaction for continuous quality improvement is sine qua non. However, policymakers must remain mindful of the potential unintended consequences if satisfaction metrics are used to evaluate healthcare professionals for performance-related decisions. To mitigate potential negative impacts on healthcare professionals, Bhutan could adopt a strategy where satisfaction surveys are used solely for organizational improvement, rather than for individual evaluations or compensation. This approach would allow healthcare providers to prioritise quality improvement without fear of punitive measures, fostering a supportive environment that upholds both patient satisfaction and provider morale.”

**Conclusion**

**Comment:** The authors could suggest areas for further research, such as longitudinal studies or comparative studies with similar healthcare systems in culturally similar countries. This would aid policymakers in anticipating and addressing shifts in patient expectations and satisfaction.

**Response:** We appreciate the reviewer’s insightful comment and have amended the document as recommended. The future research is mentioned in the conclusion between line 353-356 and it reads as follows:

“While our findings provide valuable insights, further research is necessary to identify the factors driving differences in patient satisfaction. A mixed-methods approach could uncover key contributors to these variations. Longitudinal and comparative studies across multiple hospitals could track shifts in patient expectations, while ethnographic research could reveal cultural and systemic influences. These findings could inform evidence-based policies to better address patient needs.”

**Recommendations**

**Comment:** If the paper could discuss the limitations explicitly if any would strengthen the conclusions.

**Response:** We thank the reviewer for the comment. We have included the following as a limitation of the study at the end of the discussion:

“While secondary data provides valuable insights, it also introduces certain limitations. Recall bias may have affected the accuracy of participants' responses. Additionally, for illiterate participants, responses recorded by staff or friends could have introduced errors or positive bias. Response bias, driven by factors such as literacy levels, cultural norms, or social desirability, may have influenced how participants understood and answered the survey questions. Although the survey captured all major ethnic groups in Bhutan, the proportions of respondents from each group may not align with their representation in the broader population, potentially limiting the generalizability of the findings.”

**Comment:** Qualitative follow-up with respondents could add depth to the quantitative findings, helping to contextualize why specific groups, such as the illiterate population, report higher satisfaction.

**Response:** Thank you for the insightful recommendation. We recognize the value of qualitative follow-up in providing deeper insights into patient satisfaction. However, due to the scope of this study, such follow-up would require additional research to explore the factors contributing to the higher satisfaction reported by specific groups, such as the illiterate population.

**Reviewer #3**

**Abstract:**
Good abstract
**Comment:** L32-33: you may just mention one test

**Response:** Thank you for your feedback. We have amended the abstract as recommended. It now reads:

"The data were analysed using descriptive and inferential statistics."

We opted for a general description to avoid singling out specific statistical tests, ensuring the abstract remains concise and focused while reflecting the breadth of the analysis conducted.

**Comment:** L36- mention all the ethnic groups which are significant not just others, significant by how much?

**Response:** Thank you for your comment. In this context, "Others" refers to ethnic groups not included in the three primary categories: Sharchop, Ngalop, and Lhotshampa. The survey categorised ethnicity into these three major groups and "Others" category, which encompasses minor ethnic groups that do not fall under the primary categories. As this is a retrospective analysis, the dataset does not provide further breakdowns or details on the specific ethnic groups within the "Others" category.

**Comment:** L35-41: please mention the findings in figures (% or numbers, etc) in brackets.

**Response:** Thank you for your comment. The significance levels and specific findings have been included in the relevant sections, with the overall satisfaction values presented in figures.

**Comment:** L42-46: rephrase it and make it clear, seems sentences are diluting and repeating.

**Response:** Thank you for your comment. We have amended the abstract as recommended. It now reads:

“Our findings highlight the need to address socio-demographic disparities in patient satisfaction. As the Bhutanese socio-demographic landscape evolves, satisfaction levels may decline. To enhance overall satisfaction, healthcare policymakers should focus on improving accessibility and convenience. Strategies such as establishing dynamic limits on free services, exploring private sector engagement in high-end services, and strengthening the healthcare workforce are essential for sustainable and quality healthcare service delivery.”

**Comment:** L47-49: What makes you conclude this, it should be related to your research findings not just hypothetical talk, what do you mean by high-end services, there was no finding on healthcare workforce but how come you conclude on this. Please be very specific, just mention what you get from your research.

**Response:** Thank you for your comment. Our survey analysis found that "technical quality" was one of the least satisfied domains, based on the combined responses to the four questions within this domain, which included aspects such as "service availability." This indicates that patients were generally dissatisfied with several aspects of technical quality, including the availability of services at healthcare centres. Based on these findings, we recommend reviewing the limits on free services to better manage patient expectations. Clear guidelines on service limits could help align patient expectations with the services provided. The term "dynamic" is used to emphasize the need for flexibility in service limits, which should adapt as healthcare needs and technology evolve. Similar conclusions were drawn from other survey data, where adjustments in healthcare practices may help improve overall satisfaction.

Given the potential confusion around the interpretation of "high-end services," we have revised the term to "advanced healthcare services" for clarity.

Regarding the healthcare workforce, various elements are reflected across multiple domains of the survey. For example, the "accessibility and convenience" domain, which scored one of the lowest, includes a component related to access to healthcare professionals. This is directly linked to the availability and adequacy of the healthcare workforce, which we believe plays a key role in improving accessibility and patient satisfaction. We have carefully considered all questions under the PSQ-18 and based our conclusions on these findings.

We hope this addresses your concerns and clarifies the reasoning behind our conclusions.

**Introduction:**
**General comment**- keep citation number in sequence, and so does write the flow accordingly.

**Response:** We have carefully reviewed our citations and found no errors in their numbering or sequence. However, if there are specific concerns or instances you have identified, we would greatly appreciate your guidance in addressing them.

**Comment:** L87-88: rephrase the sentence to make more urging need than just saying not utlized.

**Response:** We have carefully reviewed the mentioned line numbers but could not locate the term "not utilised." It is possible there may have been a typographical error in the line number provided. We kindly request clarification or further details to ensure we address your concern effectively.

**Comment:** L88-90: are you sure this hampers the healthcare delivery service, if yes please support by evidence.

**Response:** Currently, there is no published literature on patient satisfaction in Bhutan. This gap means policymakers and decision-makers lack documented evidence regarding which domains of quality care patients find unsatisfactory. Such evidence is essential for informed decision-making and the development of a healthcare system that is effective, efficient, and responsive to patient needs.

While healthcare managers often identify deficiencies through their experience, frequent changes in management result in these insights not being consistently retained or utilised. This lack of continuity, compounded by the absence of documented evidence, makes it challenging to address deficiencies systematically. Such a system not only hampers service delivery but also leads to a significant loss of time and resources, further straining the healthcare system.

This article is the first of its kind to scientifically highlight the need to improve key areas such as accessibility and convenience, technical quality, and the time doctors spend with patients. Without this type of evidence, addressing these issues in a sustained and structured manner becomes increasingly difficult, thereby potentially hampering healthcare service delivery.

**Materials and Methods:**
**Comment:** L94-98: Why only Mongar and Gelephu hospital, mention what is the reason for choosing these two hospitals. Why JDWNRH national referral hospital was not selected? Data says more than 60% of country's patient visit this hospital, I guess you have lost good amount of data.

**Response:** Thank you for the comment. JDWNRH has, in principle, been granted functional autonomy. As a result, the Ministry of Health's Quality Division does not directly oversee its quality-related activities. Consequently, there is no archived patient satisfaction data available for this hospital using the PSQ-18 tool.

Initially, we intended to conduct a prospective study at JDWNRH, considering its status as the nation’s apex hospital. However, we learned that a research team from KGUMSB was planning a similar study at JDWNRH using the PSQ-18 tool during the same period. To avoid duplication and overlap, we decided it would be more productive to allow these two independent studies to run concurrently. Comparing findings from both studies would provide richer and more comprehensive insights than a single study could achieve.

**Comment:** L98-99: procedure is not clear, how the patient is being enrolled. Please mention in between these two lines. Showing in a flowchart would be great though.

**Response:** Thank you for the comment. As this is a retrospective study, we initially did not provide details on how the survey was conducted. However, based on your feedback, we have now included a brief explanation of the survey procedure in the "Study Design and Setting" section. The amended section now reads as follows:

“The hospitals had previously conducted routine paper-based surveys throughout April 2024 as part of their continuous quality improvement initiatives. During this period, voluntary feedback was sought from every fifth inpatient and outpatient, aged 18 years and older. For illiterate participants, responses were recorded by their trusted friend or quality assurance officials to ensure inclusivity. The completed surveys were briefly analysed for continuous quality improvement and archived in the respective quality assurance unit.”

**Comment:** L125-126: I think you dont have to mention about the waiver for consent, this is feedback survey anyways.

**Response:** Thank you for the comment, we have removed this section.

**RESULTS:**
**Comment:** L146- are you sure about the clinical variables, I cant see in the table.

**Response:** Thank you for the opportunity to clarify. Inpatient and outpatient status are considered clinical variables because they represent distinct categories of patient care, each with unique healthcare needs and treatment approaches.

**Comment:** L148-166: Just keep few important ones, no need to describe all the variables in sentence, tables speaks. L167-168: no need to mention

**Response:** Thank you for your helpful comment. In response, we have retained only the significant variables and provided a focused description of these. The descriptions of non-significant variables have been removed, in line with your suggestion. This revision aims to enhance the clarity and conciseness of the results section, ensuring that only the most pertinent information is highlighted. We appreciate your input and believe these changes improve the manuscript.

**Comment:** Table 4: is not good table, lot of data into it and it speaks less about it. You may delete the table or modify it. Define and show the satisfaction level, how much is dissatisfied, which score is moderate and which score is satisfied, please mention either in method section or along with the table 4.
**Response**: Thank you for your valuable feedback regarding Table 4. We understand your concern about the table appearing overloaded with data, and we appreciate your suggestion to modify it. However, we believe that Table 4 provides crucial insights into patient satisfaction levels across various socio-demographic groups and domains of care, which are essential for identifying areas needing improvement.

To clarify, the patient satisfaction score is measured on a 5-point Likert scale (1-5, ranging from "Strongly Disagree" to "Strongly Agree") and it is mentioned in the methodology. The table presents the detailed distribution of scores with SD across different patient categories, offering a comprehensive view of satisfaction levels that simple percentage summaries might not fully capture. These details allow for a more nuanced understanding of satisfaction, which is vital when developing targeted strategies to improve service delivery.

For example, the outpatient domain one score of 4.10 suggests that, on average, patients "Agree" with the quality of the service delivered. Such specific interpretations across the table are instrumental in crafting focused interventions based on patient feedback, ensuring improvements are tailored to distinct patient needs.

Therefore, we would prefer to retain the detailed presentation of the findings in Table 4, as it provides essential data for evidence-based decision-making and policy formulation.

Thank you again for your insightful comment, and we hope our explanation addresses your concerns.

**DISCUSSION:**
General comment- need to refine it more clearly. Be specific with your results and support it with previous studies.

**Comment:** L194-197: rephrase the sentence, i dont think so, please support by findings, what is the proportion of the patient seen and the population of these two regions when you say it represents.

**Response:** Thank you for your insightful comment. We acknowledge the concern raised regarding the representation of patient groups in our study. Since this is a retrospective study, we did not specifically examine the proportion of patients seen from each region or the overall population demographics of these areas. However, the existing data demonstrates that all major ethnic groups in Bhutan are captured, with the Ngalop group being relatively smaller in proportion.

Unfortunately, there is limited data available on the precise population size of each ethnic group in Bhutan, as highlighted in published literature such as The Kingdom of Bhutan Health System Review. This makes it difficult to make a direct comparison between the ethnic group proportions in the study and the general population.

Therefore, with all major ethnic groups captured, we believe that the findings from this study are still relevant for national healthcare policy and decision-making.

Considering the concern raised, we have toned down the sentence, and it now reads as follows:

“The patient satisfaction survey data from MERRH and GCRRH includes patients from major Bhutanese socio-demographic groups. While the sample may not perfectly reflect the ethnic proportions of the broader population, it offers valuable insights into patient satisfaction across different groups, which can inform national healthcare policy and decision-making.”

**Comment:** L213-215: How?? need more data to support.

**Response:** Thank you for your comment. I believe you are referring to the observation that patient satisfaction declines as literacy rates improve. If I have understood your concern correctly, this study has found that literate individuals tend to report lower satisfaction compared to illiterate individuals. Therefore, as literacy levels rise, patient satisfaction may naturally decline. We have also discussed possible reasons for the lower satisfaction among literate individuals in the discussion section. For further details, please refer to the tables and the discussion.

**Comment:** Please mention the limitations and the strength of the study before the conclusion.
**Response:** Thank you for your comment. Per the suggestion the strength and specifically limitations are mentioned both in the discussion and conclusion sections.

**CONCLUSION:**
**Comment:** L334-341: no need to reflect, those are less significant to mention

**Response:** Thank you for your comment. Considering its milder significance we have removed it.

**Comment:** Please mention the overall satisfaction level of the patients in the conclusion, so that readers take away the message.

**Response:** Thank you for your comment. We have mentioned overall satisfaction and now it reads as:

“The overall patient satisfaction among Bhutanese patients is high, with a score of 4.06 on a 5-point Likert scale.”

**Reviewer #4**

Thank you for the opportunity to review this work on patient satisfaction, which is quite a young but very important indicator of quality healthcare services.
Here are my comments;

**Abstract**
**Comment:** Indicate the level of patient satisfaction as found in your study
Consider including p-values for variables that are statistically significantly associated with patient satisfaction.
**Response:** Thank you for your comment. We have mentioned the level of patient satisfaction and now it reads as:

“Overall, patient satisfaction in Bhutan is high, with an average score of 4.06 on a 5-point Likert scale. Satisfaction is particularly high in the financial domain; however, accessibility and convenience scored the lowest.”

Initially, we have used the term significance only for readability purposes. However, considering your recommendation, we have made the amendments. The sentences now read as:

“Our study revealed significant variations in patient satisfaction across socio-demographic and clinical groups. Ethnicity (P-value= 0.017), occupation (P-value= 0.014), and education level (P-value= 0.021) emerged as significant predictors of satisfaction. Sharchop and other ethnic groups (P-value= <0.001); farmers, religious personnel, and other occupational groups (P-value= <0.001); and illiterate (P-value= <0.001) individuals exhibited significantly higher satisfaction levels. While patient type (P-value= 0.472), age (P-value= 0.553), and marital status (P-value= 0.448) influenced satisfaction levels, they did not emerge as significant predictors when considering other variables. Overall, patient satisfaction in Bhutan was high, particularly in the financial domain; however, accessibility and convenience scored the lowest. Overall, patient satisfaction in Bhutan is high, with an average score of 4.06 on a 5-point Likert scale. Satisfaction is particularly high in the financial domain; however, accessibility and convenience scored the lowest.”

**Introduction.**
**Comment:** Strengths. The introduction is well structured with relevant literature capturing the concept of patient satisfaction. The authors clearly indicate the different factors that influence satisfaction and they also note that there is scarcity of published literature on the subject in Bhutan.
**Criticism & suggestions.** The researchers need to clearly indicate the role of patient satisfaction in healthcare highlighting the potential contribution of the study to practice.

**Response:** Thank you for your valuable feedback. We appreciate your observation that the introduction is well-structured and captures the concept of patient satisfaction with relevant literature.

Regarding the suggestion to clearly indicate the role of patient satisfaction in healthcare and the potential contribution of our study to practice, we have highlighted this from multiple perspectives in the introduction. For example, we have noted that patient-centered care is increasingly becoming a cornerstone of healthcare delivery, making it crucial to meet patients' needs effectively. Patient satisfaction serves as an essential metric for evaluating whether these needs are being fulfilled, thereby directly contributing to the enhancement of healthcare systems.

Furthermore, we have discussed how patient satisfaction helps identify unmet needs, providing healthcare managers and professionals with valuable insights for implementing continuous quality improvement initiatives. This linkage between patient satisfaction and quality improvement underscores the practical implications of our study in guiding evidence-based policy and practice.

We hope this clarification addresses your concern.

**Comment:** In lines 81-86, authors mention about the transition towards quality-oriented care in their setting. More literature to capture the interventions from the Bhutan authorities implemented to improve patient satisfaction should be added.

**Response:** Thank you for your insightful comment. We agree that additional literature on interventions implemented by Bhutanese authorities to improve patient satisfaction would strengthen the manuscript. While several initiatives have been undertaken by the Ministry of Health to enhance healthcare quality and safety, there is a lack of published literature documenting these efforts in a comprehensive manner.

However, we have highlighted the adoption and implementation of the Bhutan Healthcare Standard for Quality Assurance (BHSQA), which is a key initiative by the Ministry of Health to promote a quality-oriented culture in Bhutan’s healthcare system. The BHSQA, with its 116 standards, 639 objective elements, and 67 key performance indicators, forms the central framework for delivering quality and safe healthcare services across the country.

We believe that our manuscript will contribute to the literature on healthcare quality and safety in Bhutan and assist future research.

We hope this clarification addresses your concern.

**Comment:** Authors should also add more evidence regarding patient satisfaction from other settings especially preceding the description on the factors influencing satisfaction.

**Response:** Thank you for your insightful comment. We intentionally kept the discussion on patient satisfaction in the introduction broad to provide a general context. A more detailed exploration of the evidence, including a comparison and contrast of our findings with those from other settings, is included in the discussion section, where it is more appropriate to delve into such details. Therefore, in-depth evidence was not included in the introduction.

We hope this clarification addresses your concern.

**Comment:** What are different domains of satisfaction assessed in other settings and how do these assessment link into the assessments in Bhutan?

**Response:** Thank you for your insightful comment. Although the domains of patient satisfaction can vary across different settings, the seven domains in the PSQ-18 are widely recognized and frequently assessed in healthcare settings worldwide. Below are the domains of patient satisfaction in PSQ-18 and their relevance in any healthcare setting including Bhutan:

1. **General Satisfaction**: This domain assesses the overall satisfaction of patients with the healthcare services they received indicating the effectiveness of the healthcare services delivery.
2. **Technical Quality**: This domain evaluates the perceived technical competence of healthcare providers, including diagnosis accuracy, treatment appropriateness, and overall expertise.
3. **Interpersonal Manner**: This domain gauges how respectful, empathetic, and courteous healthcare providers are in their interactions with patients.
4. **Communication**: Effective communication, including clarity and attentiveness, is central to understanding patient needs and improving satisfaction.
5. **Financial Aspects**: This domain assesses patient satisfaction with the costs of care and the transparency of billing practices.
6. **Time Spent with Doctor**: This domain measures whether patients feel they have had enough time with their healthcare provider to discuss concerns and receive adequate care**.**
7. **Accessibility and Convenience**: This domain evaluates how easy it is for patients to access care, including appointment availability, waiting times, and the convenience of the healthcare facility.

These domains provide a comprehensive framework for assessing patient satisfaction and can be adapted across different healthcare settings, including those in Bhutan, to monitor and improve the quality of care.

We hope this clarification addresses your concern.

**Materials and methods**
**Comment:** The current structure of the methods section does not allow for smooth transition by the reader. The authors should consider structuring the section into subsections such as study design, study population, study setting, data collection tools, data collection procedures etc....

**Response:** Dividing the Materials and Methods section into individual sub-headings as recommended would result in a very brief and fragmented write-up, which might not effectively present the detailed methodology of the study. To ensure a more cohesive and comprehensive narrative, we have grouped related sub-headings together into four broader categories, each addressing a key aspect of the study. This approach allows us to present the methodology concisely while maintaining the clarity and flow necessary for readers to understand the process.

The grouped sub-headings are as follows:

1. Study design and setting
2. Study population and survey instrument
3. Data management and analysis
4. Ethical considerations

**Comment:** The targeted population in this study is not clear.

**Response**: Thank you for the comment. We have clarified the study population in the revised methodology section, which now reads:

“The study population consisted of patients aged 18 years and older who had voluntarily completed surveys”

**Comment:** Was the data collected secondary or primary? Lines 99-100 seem to indicate the latter but from previous statements, one could infer that the data was secondary. This needs to be clarified in your writeup. If this was secondary data, discuss the possible biases.

**Response:** Thank you for your comment. We would like to clarify that this study analyzed secondary data obtained from the patient satisfaction survey archives at MERRH and GCRRH. The confusion in lines 99-100 might have arisen due to the description of the survey methodology. While it is important to describe these details to provide context about how the data were originally collected, they do not imply that the study itself involved primary data collection. To address this, we have made amendments to the methodology at our level best to ensure it does not sound like a prospective study.

Regarding potential biases, we acknowledge that the use of secondary data may introduce limitations. Since PLOS ONE does not have a specific section for limitations, we have addressed this matter at the end of the discussion and before the conclusion, as follows:

“While secondary data provides valuable insights, it also has several limitations. As a retrospective cross-sectional design, the study cannot capture temporal changes. Recall bias may have influenced the accuracy of participants' responses. Additionally, for illiterate participants, responses recorded by staff or friends could have introduced errors or positive bias. Response bias, driven by factors such as literacy levels, cultural norms, or social desirability, may have influenced how participants understood and answered the survey questions. Although the survey captured all major ethnic groups in Bhutan, the proportions of respondents from each group may not align with their representation in the broader population, potentially limiting the generalizability of the findings.”

We hope these changes clarify the concern and improve the clarity of our study's methodology.

**Comment:** The authors need to indicate the time points/periods from which the data was obtained. Do you think this affects satisfaction?

**Response:** Thank you for your comment. In the materials and methods section, we have clearly stated that the original survey was conducted throughout April 2024, and the archived data was accessed on 15th July 2024. In our opinion, the time points do not significantly impact the satisfaction levels, as there has been no fluctuation in the hospital's service delivery during this period. Furthermore, there were no reports of significant population changes (either influx or efflux) in the area that could have influenced the data.

**Comment:** If this was secondary data, did the authors design a data abstraction?

**Response:** Thank you for your comment. Since the data were archived and already organized in the original survey, no further abstraction was necessary. The information was analyzed as is, directly from the survey responses, which included socio-demographic details and satisfaction indicators. Therefore, no additional data abstraction was performed beyond the retrieval and analysis of the existing dataset.

**Comment:** Was this informed by the PSQ18? Has the PSQ18 been utilized in other research studies? Is it a valid and reliable tool for establishing patient satisfaction? What was the internal consistency in this current study? The researchers should provide the tool used in this assessment as a supplementary material for replicability of the study in other settings.

**Response:** Thank you for your comment. Yes, the study was informed by the PSQ-18 framework. The only changes made were the inclusion of sociodemographic information and the replacement of terms like "doctor" or "doctor's office" with more inclusive terms such as "healthcare professional" or "healthcare centre" to ensure broader applicability across various healthcare settings. The domains, specific questions, and scoring system, including the re-scaling, followed the PSQ-18 standard, and these aspects are reflected in the methodology section.

The PSQ-18 is a well-established and widely used tool for measuring patient satisfaction, with established internal consistency as reported in its original publication. Given that this study is a retrospective analysis with only one response per patient, we did not assess internal consistency. The tool's reliability has already been demonstrated in previous studies, and we utilized it as is, in line with its established use in various healthcare settings.

The PSQ-18 is a widely recognized and validated tool, and we have not made significant modifications to its original structure, as clarified previously. Therefore, we believe that providing the full tool as supplementary material does not add significant value to the article, nor does it impact the ability to replicate the study in the future. The tool's content is well-established and available in the literature, and we have adhered closely to its original form.

We hope this clarification addresses your concern.

**Comment:** Line 177 describes how the researchers quantified patient satisfaction by getting the average scores across 7 domains of the tool. Is this the standard approach or this was adapted?

**Response:** Thank you for your comment. I believe you are referring to line 117, not 177. Calculating an overall satisfaction score by averaging the scores across all domains of the PSQ-18 is a common and logical approach, as evidenced by its use in various published studies. This method aligns with the conceptual framework of the tool, where the domains collectively represent different facets of patient satisfaction. Patients typically experience multiple domains of care during a single visit, and their overall satisfaction reflects the cumulative impact of these experiences. By averaging the scores, we provide a concise and representative measure of overall satisfaction.

We hope this clarification addresses your concern.

**Results**
**Comment:** Presenting the results with subsections as per the meaning of the findings would improve the flow of information in this section.

**Response:** Response: Thank you for your comment. While your suggestion is valuable, we believe that the concern is about the presentation of domain-specific results at the end of the results section. We have structured the results in the following sequence based on the flow of analysis and the specific focus of this article:

1. Sociodemographic profile
2. Difference in patient satisfaction among different sociodemographic and clinical variables and subgroups
3. Predictors of patient satisfaction
4. Patient satisfaction in individual domains of PSQ-18

We placed the domain-specific results last because presenting them earlier, before conducting ANOVA and Dunnett's grouping, would require computing satisfaction levels for 33 separate subgroups. This approach would not allow us to merge insignificant groups, potentially leading to less meaningful interpretations. By performing the domain-specific analysis after the ANOVA, we were able to group subgroups into 16 and present more meaningful, consolidated data. This approach aligns with the goals of our analysis and improves the clarity of the results.

We hope this clarification addresses your concern.

**Comment:** Author need to revise the use of some words such as control group, comparison group as these could mean different parameters in different study designs.

**Response:** Thank you for your comment. I believe your concern refers to the terminology used in the ANOVA section of the results. In this study, the term "comparison" is used in the context of Dunnett's test, which compares multiple treatment groups to a single control group. The "control group" specifically refers to the group that serves as a reference or baseline for comparison. These terms are standard in the context of Dunnett’s test for multiple comparisons, where the control group is the benchmark, and other groups are compared against it. We hope this clarification addresses your concern.

**Comment:** Were the odds ratios presented in table 3 crude or adjusted? You need to consider including both.

**Response:** We sincerely thank the reviewer for their insightful comment. We would like to clarify that the odds ratios presented in Table 3 are indeed adjusted odds ratios (aOR).

Patient satisfaction is a complex and multifactorial outcome, influenced by various sociodemographic and clinical factors. Crude odds ratios (cOR), while useful in exploring initial relationships, might provide misleading information by failing to control for confounding variables. For instance, a variable might seem significantly associated with patient satisfaction in a crude analysis, but once adjusted for other variables, the association may weaken or disappear. Conversely, some relationships may only emerge after accounting for potential confounders.

Therefore, in our study, adjusted odds ratios (aOR) offer a more accurate and meaningful understanding of the predictors of patient satisfaction, reflecting the real-world influence of various interrelated factors. For clarity, we have revised Table 3 to explicitly label the odds ratios as "Adjusted Odds Ratios" to avoid any confusion. We hope this clarification addresses your concern.

**Comment:** According to the authors, the variables ethnic group and occupation were significantly associated with satisfaction. What was the rationale for creating the categories under these two variables? Are the individuals under the same group having similar characterization? and if not, could it be a biased assignment? For instance, in the category of occupation, why are students in the same group as civil servants?

**Response:** We sincerely thank the reviewer for their thoughtful comment regarding categorising ethnic groups and occupations in our study. As this is a retrospective analysis, the ethnic groups included in the study are those captured in the original survey. According to our knowledge and supported by existing literature, the primary ethnic groups in Bhutan are Sharchop, Lhotshampa, and Ngalops. The categories of ethnic groups captured by the survey reflect the common ethnic groups within the study population and are designed to align with the major sociocultural distinctions in Bhutan.

We acknowledge that ethnic groups may exhibit varying characteristics beyond the classification used in our study. However, the current groupings are based on major sociocultural and historical distinctions that are meaningful in the context of Bhutan’s population. Therefore, we believe this approach is appropriate for our study, given the constraints of the available data.

Thank you for your insightful comment regarding the occupation variable. As mentioned earlier, the occupational groups presented in the study reflect those captured by the original survey, which categorized individuals based on their current roles within society. As with many surveys, students are classified under occupation to provide a clear demographic structure.

While students and civil servants may have distinct characteristics, the original survey grouping aimed to balance simplicity with statistical power. Additionally, our statistical analysis, including Dunnett’s multiple comparisons and other grouping systems, did not find significant differences between students and the other occupational groups, suggesting that the broad classification used in the original survey is appropriate for this study's context.

We hope this clarification addresses your concern.

**Comment:** Based on your analysis plan, you need to indicate in the results section the proportion of those patients who are satisfied and dissatisfied.

**Response:** Thank you for your comment. The dichotomization of respondents into "satisfied" and "dissatisfied" was undertaken specifically for regression analysis. The mean satisfaction scores and standard deviations presented in Table 4 offer a more detailed and nuanced reflection of patient satisfaction across various domains and demographics.

We believe that a simple percentage summary of "satisfied" versus "dissatisfied" might oversimplify the findings, failing to provide the comprehensive view necessary to inform actionable strategies. Table 4 highlights the detailed distribution of scores, which helps uncover subtle variations in satisfaction levels among different patient groups. These insights are crucial for developing targeted interventions aimed at enhancing service delivery and meeting the specific needs of patients. We hope this explanation clarifies our rationale for presenting the data in its current format.

**Discussion**
**Comment:** The researchers have done a great job in discussing and linking their findings to the already existing body of knowledge and the implications of the findings are clear. This section can be improved by;
Providing a detailed discussion of the limitations associated with methodological weaknesses, highlighting their impact on the generalizability of the study findings.

**Response:** Thank you for the comment. PLOS ONE does not have a specific section for limitations, we have addressed this matter at the end of the discussion and before the conclusion, as follows:

“While secondary data provides valuable insights, it also has several limitations. As a retrospective cross-sectional design, the study cannot capture temporal changes. Recall bias may have influenced the accuracy of participants' responses. Additionally, for illiterate participants, responses recorded by staff or friends could have introduced errors or positive bias. Response bias, driven by factors such as literacy levels, cultural norms, or social desirability, may have influenced how participants understood and answered the survey questions. Although the survey captured all major ethnic groups in Bhutan, the proportions of respondents from each group may not align with their representation in the broader population, potentially limiting the generalizability of the findings.”

**Comment:** The structuring of the discussion should be changed. The level of satisfaction should be discussed first followed by the factors associated with satisfaction.

**Response:** Thank you for your comment. As addressed in the earlier section, there is a slight difference in how the results are presented due to the focus of the study and the sequence of the analysis. In the discussion, we followed the same sequence as the results section to maintain consistency and coherence in presenting the findings. We hope this explanation clarifies our rationale for presenting the data in its current format.

**Reviewer #5**

**Comment:** I feel that the paper is well written and its findings are very significant to health policy maker and also for the country, this study will serve as a baseline on patient satisfaction level for future researches.

**Response:** Thank you for your positive feedback.

**Reviewer #6**

This manuscript presents a timely and relevant study on patient satisfaction in Bhutan, a country with a developing healthcare system. The study's findings, particularly the identification of ethnicity, occupation, and education level as significant predictors of patient satisfaction, offer valuable insights for healthcare policymakers and professionals in Bhutan and other countries with similar healthcare systems.

Strengths
The research addresses the important question of patient satisfaction within the Bhutanese healthcare system, which is undergoing a transition towards a quality-oriented culture. This is particularly relevant given the global emphasis on patient-centered care and the need to understand the factors that influence patient satisfaction in diverse healthcare settings.
The study adds to the existing body of literature by providing empirical evidence on patient satisfaction within the specific context of Bhutan. This is a valuable contribution as there is limited research on this topic in Bhutan, and the study's findings offer unique insights into the factors that influence patient satisfaction in a developing healthcare system
The conclusions are consistent with the evidence and arguments presented in the study. The authors have accurately summarized the key findings and their implications for healthcare policy and practice

**Areas for Improvement**
**Comment:** The authors mention the influence of Bhutan's cultural context on patient satisfaction, they could expand this discussion to provide a more nuanced understanding of how cultural factors, such as the emphasis on compassion and the role of Buddhism, may influence patient expectations and perceptions of healthcare services.

**Response:** Thank you for your insightful comment. As recommended, we have expanded the discussion to provide a more nuanced understanding. The revised section now reads:

“Additionally, Bhutan’s culture is deeply influenced by Buddhism in all aspects of life. Buddhist philosophy, which emphasizes compassion, kindness, and non-violence, shapes the values and behaviours of the Bhutanese people. These cultural values profoundly affect how patients perceive and respond to healthcare services. In Bhutan, there is often a tendency to provide positive feedback, even when the service does not fully meet expectations. This may stem from cultural norms that prioritize harmony and respect, which are integral to Buddhist teachings. As a result, patients may focus on expressing gratitude and appreciation for healthcare providers, sometimes refraining from negative feedback to maintain social harmony.”

**Comment:** “This study retrospectively analysed patient satisfaction survey responses collected from Mongar Eastern Regional Referral Hospital (MERRH) and Gelephu Central Regional Referral Hospital (GCRRH). The routine paper-based surveys were conducted throughout April 2024.” The manuscript lacks a detailed description of the data collection process, including the sampling method. Providing this information would strengthen the study's methodology and enhance the credibility of the findings

**Response:** Thank you for your insightful comment. We acknowledge that we failed to mention that all the archived data were collected from these two centres. Since this is a retrospective study and all the data were retrieved, there is no need for an exhaustive description of the data collection process and sampling methods. However, considering the recommendations made by you and other reviewers, we have amended the entire Materials and Methods section to address the relevant and pertinent concerns raised. The methodology is now organised into the following sections:

1. Study design and setting
2. Study population and survey instrument
3. Data management and analysis
4. Ethical considerations

**Comment:** The manuscript could benefit from more specific and actionable recommendations for healthcare policymakers and professionals. For instance, the authors could provide concrete examples of targeted interventions to address the anticipated decline in patient satisfaction due to changing socio-demographic characteristics.

**Response:** Thank you for the comment. We acknowledge the importance of providing specific and actionable recommendations for healthcare policymakers and professionals. However, in our context, numerous minor interventions could be implemented, and explicitly recommending each one after identifying it as a potential cause of low satisfaction might make the text overly wordy. Instead, we have focused on key actionable interventions, such as establishing dynamic limits on free services, exploring private sector engagement in advanced healthcare services, and strengthening the healthcare workforce.

For instance, we reported that educated individuals tend to report lower satisfaction, potentially due to exposure to stricter quality standards. The targeted intervention implied here is the adoption and implementation of international quality standards to improve patient satisfaction. Similarly, we reported that elderly patients report higher satisfaction, possibly due to patient-centric engagement practices by healthcare professionals. This implies the need to enhance healthcare professionals' skills in providing patient-centric care for all as a targeted intervention.

We hope this explanation clarifies our rationale for presenting the manuscript in its current format.

**Writing Quality**
The paper is well-written, with clear and easy-to-read text. The authors have used appropriate language and style for an academic audience.

**Reviewer #7**

**General Comments**
Dorji K et al. attempted to provide valuable insights into patient satisfaction levels at two referral hospitals in Bhutan, using retrospective data and exploring socio-demographic predictors of satisfaction. This research addresses an important gap in understanding healthcare quality in Bhutan. However, there are areas where further clarity would strengthen the findings. Enhancing transparency in the methodology, particularly around sample size, sampling methods, and survey administration, would strengthen this study. Addressing potential biases and improving the contextual richness of the discussion would also benefit readers and support the study’s practical implications for healthcare policy in Bhutan.

**Abstract**
**Comment:** Specificity in reporting results (Lines 22-49): The abstract provides a clear summary but could benefit from additional specificity. Including exact figures for satisfaction levels or effect sizes of significant predictors would make the results more informative for the reader.

**Response:** Thank you for your comment. The significance levels and specific findings have been included in the relevant sections, with the overall satisfaction values presented in figures.

**Introduction**
**Comment:** Bhutan’s health system (Lines 69-70): While the introduction discusses patient satisfaction broadly, it could be enhanced by offering more context on Bhutan’s healthcare system. For e.g., more emphasis on free healthcare, and the challenges associated with rural accessibility and workforce shortages would provide readers with a clearer understanding of why patient satisfaction is critical in this setting.

**Response:** Thank you for your comment. As recommended, we have briefly touched on the topic. The amended introduction section reads as follows:

“Over the years, Bhutan’s healthcare system has made remarkable progress; however, it continues to face substantial challenges in delivering quality and safe services. These include overburdened staff, shortages of specialised professionals, limited financial resources restricting access to advanced equipment and medicines, and inadequate infrastructure. Additionally, geographical barriers such as rugged terrain and poor road connectivity further hinder access, especially in remote areas [12–15].”

**Comment:** Cite BHSQA (Lines 75-76)

**Response:** Thank you for your comment. The available reference is provided.

**Materials and Methods
Comment:** Study design and sampling method (Lines 93-128): Clarify the sampling method, especially whether it was a convenience sample or if random sampling was applied. Sampling methods are essential to understanding potential biases, as convenience sampling can limit generalizability. The use of a retrospective cross-sectional design is appropriate for the data available, but its limitations should be noted, as this design cannot capture temporal changes in satisfaction or causality. Future studies could benefit from a longitudinal approach to observe changes in satisfaction over time, especially with healthcare reforms.

**Response:** Thank you for your comment. We have mentioned the sampling process and it reads as:

“During this period, voluntary feedback was sought from every fifth inpatient and outpatient, aged 18 years and older. The completed surveys were briefly analysed for continuous quality improvement and archived in the respective quality assurance unit.”

The study design is addressed as recommended and it reads as:

“This retrospective cross-sectional study analysed archived patient satisfaction survey data collected from Mongar Eastern Regional Referral Hospital (MERRH) and Gelephu Central Regional Referral Hospital (GCRRH).”

The limitation of the study is mentioned in the end of the discussion and it reads as:

“While secondary data provides valuable insights, it also has several limitations. As a retrospective cross-sectional design, the study cannot capture temporal changes. Recall bias may have influenced the accuracy of participants' responses. Additionally, for illiterate participants, responses recorded by staff or friends could have introduced errors or positive bias. Response bias, driven by factors such as literacy levels, cultural norms, or social desirability, may have influenced how participants understood and answered the survey questions. Although the survey captured all major ethnic groups in Bhutan, the proportions of respondents from each group may not align with their representation in the broader population, potentially limiting the generalizability of the findings.”

Furthermore, further research is recommended in the conclusion section which reads as:

“While our findings provide valuable insights, further research is necessary to identify the factors driving differences in patient satisfaction. A mixed-methods approach could uncover key contributors to these variations. Longitudinal and comparative studies across multiple hospitals could track shifts in patient expectations, while ethnographic research could reveal cultural and systemic influences. These findings could inform evidence-based policies to better address patient needs.”

**Comment:** Inclusion of western region hospitals (Lines 93-128): The study’s sample is limited to hospitals in eastern and southern Bhutan, which may not capture the full demographic diversity of the population. Including a referral hospital from the western region, such as JDWNRH, would enhance representativeness, as patients in the west may have different expectations and healthcare perceptions.

**Response:** Thank you for the comment. JDWNRH has been granted functional autonomy, and as a result, the Ministry of Health's Quality Division does not directly oversee quality activities at JDWNRH. Therefore, there is no archived patient satisfaction data using the PSQ-18 tool available for this hospital.

Considering JDWNRH is the apex hospital of the nation, we initially intended to conduct a prospective study there. However, we came to know that a team of researchers from KGUMSB was already planning a prospective patient satisfaction study at JDWNRH using the PSQ-18 during the same period. Given this, we felt it would be more beneficial to allow these two independent studies to run concurrently and compare their findings, as this would provide more valuable insights than conducting a single study.

We hope this explanation clarifies our rationale for not including JDWNRH.

**Comment:** Mode of survey administration (Lines 99-100): If health professionals conducted surveys for illiterate patients, this could introduce interviewer bias, leading patients to feel pressured to respond positively. The manuscript should clarify who administered the surveys and acknowledge any potential interviewer effects. A more consistent survey administration mode, such as anonymous or third-party administration, could reduce response bias, especially among illiterate respondents.

**Response:** Thank you for your comment. We have provided detailed information regarding the survey administration process under the study design and setting section of the materials and methods. Additionally, the limitations of the survey methodology have been acknowledged, and recommendations for adopting online and anonymous survey systems or engaging external evaluators have been discussed in the Discussion section.

**Comment:** Sample size (Lines 93-128): The study does not provide a power analysis or sample size calculation. Although 915 respondents seem more than adequate, a statistical justification would add methodological correctness. Reporting a power analysis based on effect sizes would strengthen the credibility of the sample size in capturing true effects across socio-demographic groups.

**Response:** Thank you for your thoughtful comment. As this is a retrospective cross-sectional study, all available data from the archived surveys were retrieved and utilised. Since the study is based on an existing dataset rather than a prospective design, a priori sample size calculation was not applicable in this context.

However, per your recommendation, we calculated the effect size estimates using Cohen's d or f to quantify the magnitude of group differences. Additionally, a post-hoc power analysis was conducted to evaluate the adequacy of the sample size in detecting meaningful effects. These findings have been incorporated into Table 2, and the methodology has been updated under the data management and analysis section accordingly.

**Comment:** The method could benefit from greater transparency regarding the survey modification process for PSQ-18. Provide additional information on the survey's adaptation process and provide the modified PSQ-18 as supplementary file (Lines 100-107).

**Response:** The modification has been detailed in the methodology section. There has been no major modification to the PSQ-18 questionnaire; the only changes are the replacement of the terms "doctor" and "doctor’s office" with "healthcare professionals" and "healthcare centre," respectively. These changes were made to ensure broader applicability across healthcare settings while maintaining the original intent of the questions. To avoid confusion, the term "modified version" has been replaced with "adapted version" to reflect that the original intent of the questions was preserved.

Since the changes are minimal and do not affect the questionnaire’s original structure, format, or intent, we have not conducted a formal assessment of the adapted tool’s reliability or validity in this new context. Given that this is a retrospective study, performing such an assessment was not feasible. Additionally, as the PSQ-18 is a well-established instrument, we believe that providing the adapted version as a supplementary file would not add significant value to the manuscript.

We hope this explanation clarifies your concerns.

**Comment:** Multiple comparisons correction in ANOVA (Lines 114-115): The authors used one-way ANOVA with Dunnett grouping for multiple comparisons across socio-demographic groups, but it lacks a correction for multiple comparisons. Given the numerous factors compared, using a method like Bonferroni or Holm correction would reduce the likelihood of Type I error. This correction would ensure that significant differences between groups are robust and not due to chance alone.

**Response:** Thank you for the thoughtful comment. We appreciate your concern regarding the correction for multiple comparisons. In our study, we employed Dunnett’s method for multiple comparisons, which is specifically designed to control the familywise error rate (FWER) and, thereby, control the rate of Type I errors. Dunnett's method adjusts the error rate for individual comparisons (individual error rate), based on the chosen family error rate.

We believe that this approach is suitable for our analysis, where comparisons were made between multiple groups and a reference group. As Dunnett’s method inherently controls for the multiple comparisons in this context, additional corrections, such as Bonferroni or Holm, were not applied. Thank you again for your valuable suggestion. We hope this explanation clarifies our approach.

**Comment:** Significance levels (Line 121): The manuscript uses a significance level of p < 0.005, which is stricter than the conventional p < 0.05. This could strengthen findings by reducing the risk of Type I errors, but it would be good to clarify why this threshold was chosen. If a multiple-comparisons correction (such as the Bonferroni correction) was applied to account for repeated testing across multiple domains, mentioning this would add more detail.
Results

**Response:** Thank you for your comment. The significance level used in our study is p < 0.05, as is standard practice in most studies. The reference to p < 0.005 was a typographical error, and this has been corrected in the manuscript. We appreciate your attention to detail, and the revised version now reflects the correct significance threshold.

**Comment:** Visual summaries (Lines 129-187): While the tables are comprehensive, adding visualizations (e.g., bar charts or histograms) for key socio-demographic variables and satisfaction levels across domains would improve accessibility and make trends easier to interpret. These visuals could also help in identifying outliers or skewness that might affect parametric assumptions in ANOVA.

**Response:** We appreciate the reviewer’s insightful suggestion. Visual summaries, such as bar charts or histograms, could indeed enhance accessibility and facilitate interpretation of key trends. From the outset, we considered incorporating visual representations; however, due to the exhaustive nature of the data, we encountered challenges in doing so without leading to redundancy. While the tables are crucial for detailed presentation, extracting key findings from them to create visual representations would result in a duplication of information. Given the length of the article, we believe that the tables alone provide sufficient clarity for effective communication of the results. We hope this explanation addresses the reviewer’s concern, and we remain open to further feedback.

**Comment:** Wide confidence intervals in logistic regression (Lines 171-175): Some odds ratios show wide confidence intervals, indicating limited precision, possibly due to small subgroup sizes or inherent variability. Briefly acknowledging this limitation and its potential impact on the reliability of estimates would add transparency to the findings.

**Response:** We appreciate the reviewer’s comment regarding the wide confidence intervals (CIs) for some of the odds ratios in our logistic regression analysis. We acknowledge that wide CIs can indicate limited precision in the estimates, which may be due to factors such as small subgroup sizes or variability within certain categories.

In particular, the illiterate group, with 238 participants, has a broader confidence interval, which likely contributes to the observed imprecision. Similarly, certain demographic and occupational subgroups may exhibit higher variability, impacting the precision of estimates in those categories.

To improve transparency and address this limitation, we have included a brief mention of this issue in the **Discussion** section, acknowledging that it could be due to the inherent varibillity or effect of sample size.

**Discussion**

**Comment:** Cultural factors and contextual influences on satisfaction: The discussion effectively ties findings to existing literature but could benefit from a more in-depth look at Bhutan-specific cultural factors that might influence satisfaction. For instance, the cultural respect for authority figures and the potential for social desirability bias in survey responses could influence satisfaction ratings, particularly among illiterate respondents.

**Response:** Thank you for your insightful comment. We appreciate the opportunity to deepen the discussion with additional cultural and contextual insights.

In our initial manuscript, we briefly touched upon the influence of Bhutan’s cultural values on patient satisfaction. Based on your suggestion, we have expanded this section to better reflect the cultural and social dynamics at play. We have added the following to the discussion:

"Bhutan’s culture is deeply influenced by Buddhism in all aspects of life. Buddhist philosophy, which emphasizes compassion, kindness, and non-violence, shapes the values and behaviours of the Bhutanese people. These cultural values profoundly affect how patients perceive and respond to healthcare services. In Bhutan, there is often a tendency to provide positive feedback, even when the service does not fully meet expectations. This may stem from cultural norms that prioritize harmony and respect, which are integral to Buddhist teachings. As a result, patients may focus on expressing gratitude and appreciation for healthcare providers, sometimes refraining from negative feedback to maintain social harmony."

Additionally, we have acknowledged the impact of illiteracy on patient satisfaction as a limitation at the end of the discussion, recognizing its potential influence on survey responses and how it may affect the overall findings.

**Comment:** Explanation of higher satisfaction among illiterate participants (Lines 199-218): The significantly higher satisfaction among illiterate patients is noteworthy but could partly reflect interviewer influence if surveys were administered by healthcare professionals. Discussing this potential interviewer effect here would provide insight into how survey mode might impact satisfaction scores across education levels.

**Response:** Thank you for the insightful comment. In the relevant section, we have focused on exploring why educated individuals might have expressed lower satisfaction. Regarding illiterate individuals, we acknowledge this as a limitation and have addressed it at the end of the discussion. The section now reads as:

“Additionally, for illiterate participants, responses recorded by staff or friends could have introduced errors or positive bias. Response bias, driven by factors such as literacy levels, cultural norms, or social desirability, may have influenced how participants understood and answered the survey questions.”

**Comment:** Limitations in regional representation (Lines 188-327): The findings from Mongar ERRH and Gelephu CRRH may not fully represent national patient satisfaction trends in Bhutan, as the absence of data from the west (e.g., Thimphu) could exclude urban patient perspectives and include more representation of ngalops. Acknowledge this limitation in the discussion and suggest that future research include hospitals from multiple regions for a more balanced national representation.

**Response:** Thank you for the insightful comment. We have essentially addressed the concerns raised by adding limitations at the end of the discussion section. It reads as:

“While secondary data provides valuable insights, it also has several limitations. As a retrospective cross-sectional design, the study cannot capture temporal changes. Recall bias may have influenced the accuracy of participants' responses. Additionally, for illiterate participants, responses recorded by staff or friends could have introduced errors or positive bias. Response bias, driven by factors such as literacy levels, cultural norms, or social desirability, may have influenced how participants understood and answered the survey questions. Although the survey captured all major ethnic groups in Bhutan, the proportions of respondents from each group may not align with their representation in the broader population, potentially limiting the generalizability of the findings.”

Additionally, the necessity of future research is mentioned in the conclusion section:

“While our findings provide valuable insights, further research is necessary to identify the factors driving differences in patient satisfaction. A mixed-methods approach could uncover key contributors to these variations. Longitudinal and comparative studies across multiple hospitals could track shifts in patient expectations, while ethnographic research could reveal cultural and systemic influences. These findings could inform evidence-based policies to better address patient needs.”

**Comment:** Policy implications and recommendations (Lines 278-350): The authors have provided valuable recommendations for improving patient satisfaction including enhancing accessibility and convenience. Strengthening this section with specific, immediate actions, for e.g., implementing a pilot program for anonymous feedback collection, could make the policy implications more actionable for health administrators in Bhutan.

**Response:** Thank you for the comment. We added the following in the discussion section:

"Notably, in our context, the systematic collection of end-user feedback is limited. As a result, strategies may sometimes lack alignment with the actual needs and expectations of the population. Therefore, piloting an online system for anonymous feedback collection could help policymakers develop strategies that better address the needs of health service users."
