# Supplementary material for: Patient satisfaction in regional referral hospitals of Bhutan: Insights from a cross-sectional study
Source: PLoS One. 2025 Jun 2;20(6):e0312629. doi: 10.1371/journal.pone.0312629 (PMC12129210; doi:10.1371/journal.pone.0312629)
Supplement: Attachment — Submitted filename: Reviewers comment.docx [file pone.0312629.s003.docx]

**Title:** Patient satisfaction in regional referral hospitals of Bhutan: Insights from a cross-sectional study

- Well-structured, relevant, and methodologically sound.
- Strong use of statistical analysis to determine patient satisfaction predictors.

**Areas for Improvement**

A. **Introduction**

Line 61-62

“However, there is still no consensus on a universally accepted definition of patient satisfaction.”

Comment: What definition was opted for patient satisfaction assessment in Bhutan’s Context for this study?

Line 74-92

**Comment:** The discussion on Bhutan’s healthcare system is informative, but a comparison with other low-resource settings would strengthen the rationale. The introduction does not clearly explain why this study is particularly needed in Bhutan.

B. **Methods**

Line 111-114

1) “During this period, voluntary feedback was sought from every fifth inpatient and outpatient, aged 18 years and older.”

Comment: Why was every fifth patient chosen? A justification for this method (e.g., randomization, reducing response bias) would strengthen the methodology.

2) “For illiterate participants, responses were recorded by their friends or quality assurance officials.” This introduces possible response bias since friends or officials may influence responses.

Comment: Discuss whether any measures were taken to mitigate this bias (e.g., using neutral interviewers).

**C. Discussion**

Line 239 - 251

"Similarly, educated populations, particularly those with exposure to superior services and familiarity with stringent quality standards, may harbor elevated expectations, potentially resulting in lower satisfaction levels."

Comment: Other factors (e.g., hospital workload, type of medical condition) might also affect satisfaction but are not discussed.

Suggestion: Acknowledge potential confounders and their limitations in interpreting results.

**D. Conclusion**

Line 408 - 409

“As Bhutan's healthcare system evolves, leveraging patient satisfaction data for CQI is crucial.”

This is a general statement that could be more impactful.

Comment: End with a call to action, such as recommending regular nationwide patient satisfaction surveys.

**Additional Recommendations**

- Ensure all tables and figures are clearly referenced in the text.
- Maintain consistency in citation formatting (some references use "et al." while others list all authors).
