# Supplementary material for: Patient satisfaction in regional referral hospitals of Bhutan: Insights from a cross-sectional study
Source: PLoS One. 2025 Jun 2;20(6):e0312629. doi: 10.1371/journal.pone.0312629 (PMC12129210; doi:10.1371/journal.pone.0312629)
Supplement: Attachment — Submitted filename: Response_to_Reviewers_auresp_2.docx [file pone.0312629.s006.docx]

**Response to Reviewers’ Comments (email Attachment)**

**Title:** Patient satisfaction in regional referral hospitals of Bhutan: Insights from a cross-sectional study

- Well-structured, relevant, and methodologically sound.
- Strong use of statistical analysis to determine patient satisfaction predictors.

**Areas for Improvement**

**A. Introduction**

**Comment:** Line 61-62

“However, there is still no consensus on a universally accepted definition of patient satisfaction.”

Comment: What definition was opted for patient satisfaction assessment in Bhutan’s Context for this study?

**Response:** We sincerely thank the reviewer for their insightful comment. As stated in the manuscript, there is no universally accepted definition of patient satisfaction, as it is multifactorial and subjective. Therefore, this study did not adopt any specific definition. Instead, patient satisfaction was assessed using the Patient Satisfaction Questionnaire Short Form (PSQ-18), a validated and widely used survey tool that measures key domains such as general satisfaction, technical quality, interpersonal manners, communication, financial aspects, time spent with the doctor, and accessibility/convenience. By using this standardized instrument, the study ensured an objective and structured evaluation without the need for an independent definition, facilitating a more practical and comparable assessment in line with established methodologies in patient satisfaction research.

Line 74-92

**Comment:** The discussion on Bhutan’s healthcare system is informative, but a comparison with other low-resource settings would strengthen the rationale. The introduction does not clearly explain why this study is particularly needed in Bhutan.

**Response:** We sincerely thank the reviewer for their insightful comment. We chose not to include comparisons with other low-resource settings in the introduction, as we believe such comparisons are more appropriate for the discussion section. Instead, we focused on emphasizing the broader significance of measuring patient satisfaction to establish a strong rationale for this study.

While patient satisfaction evaluation is essential in all healthcare systems globally, the Bhutanese healthcare system is no exception. Assessing customer satisfaction is a fundamental component of continuous quality improvement in any service-oriented sector. Although there is no uniquely urgent need for patient satisfaction studies in Bhutan, we have mentioned in manuscript that the country is in the early stages of developing a quality-oriented healthcare culture. Bhutan has adopted the Bhutan Healthcare Standard for Quality Assurance (BHSQA) as its national healthcare standard, with patient satisfaction as one of its key performance indicators. While other indicators are also important, evaluating patient satisfaction at this foundational stage provides valuable insights into service delivery and areas for improvement, supporting the country's broader efforts to enhance healthcare quality. We hope this clarifies our approach.

**B. Methods**

Line 111-114

1) “During this period, voluntary feedback was sought from every fifth inpatient and outpatient, aged 18 years and older.”

Comment: Why was every fifth patient chosen? A justification for this method (e.g., randomization, reducing response bias) would strengthen the methodology.

**Response:** We sincerely thank the reviewer for their insightful comment. As this is a retrospective study, the researchers did not have control over the sampling approach. The survey was part of the hospital’s routine quality assessment, and the sampling method was predetermined by the respective hospitals. The hospitals selected every fifth patient to meet their predefined sample size requirements, which were primarily influenced by patient load, the need for systematic selection, and efforts to reduce selection bias.

We have revised the methodology section accordingly and added the following statement to reflect the hospital’s actual intention:

“A systematic selection of every fifth patient was employed to minimize selection bias and meet the hospital's predefined sample size requirements for internal evaluation”

2) “For illiterate participants, responses were recorded by their friends or quality assurance officials.” This introduces possible response bias since friends or officials may influence responses.

Comment: Discuss whether any measures were taken to mitigate this bias (e.g., using neutral interviewers).

**Response:** We sincerely thank the reviewer for their valuable comment. We acknowledge that the actual survey conducted by the hospitals did not include specific measures to mitigate response bias. As mentioned earlier in the manuscript, since the survey was administered by the hospitals, it could have introduced a response bias, particularly skewed toward positive responses. To address this, we suggested the implementation of online survey systems or the engagement of external evaluators.

In response to the additional concern raised by the reviewer regarding the potential influence of friends or quality assurance officials when recording responses for illiterate participants, we have amended the discussion section as follows:

“However, it is important to acknowledge that the surveys were administered by the quality units of the respective healthcare centres, and for illiterate participants, responses were recorded with the assistance of friends or quality assurance officials. This process may have introduced response bias. To mitigate this, healthcare centres could consider implementing online, anonymous survey systems or engaging external evaluators to assess patient satisfaction.

We hope this amendment addresses the concern raised.

**C. Discussion**

Line 239 - 251

"Similarly, educated populations, particularly those with exposure to superior services and familiarity with stringent quality standards, may harbor elevated expectations, potentially resulting in lower satisfaction levels."

Comment: Other factors (e.g., hospital workload, type of medical condition) might also affect satisfaction but are not discussed.

Suggestion: Acknowledge potential confounders and their limitations in interpreting results.

**Response:** We sincerely thank the reviewer for their insightful comment. Patient satisfaction is a multifactorial concept, and we have emphasized this throughout the manuscript. In our discussion, we focused on the significant predictors identified through multivariate logistic regression, which accounts for confounding factors. Therefore, we did not specifically discuss confounders in relation to education.

Additionally, we highlighted that factors such as patient type, age, sex, and marital status were not significant predictors after adjusting for other variables, underscoring the importance of considering confounding factors.

We hope this response clarifies our reasoning for not explicitly discussing confounding factors in relation to education

**D. Conclusion**

Line 408 - 409

“As Bhutan's healthcare system evolves, leveraging patient satisfaction data for CQI is crucial.”

This is a general statement that could be more impactful.

Comment: End with a call to action, such as recommending regular nationwide patient satisfaction surveys.

Response: We sincerely appreciate the reviewer’s insightful comment. We have revised the statement as follows:

“As Bhutan's healthcare system evolves, patient satisfaction data should be systematically integrated into CQI efforts. Regular nationwide assessments must be implemented to identify areas for improvement while ensuring a balanced approach to safeguard healthcare professionals' morale and care standards”

**Additional Recommendations**

- Ensure all tables and figures are clearly referenced in the text.

Response: Thank you for the comment, we have again checked ensured that tables and figures are clearly referenced in the text.

- Maintain consistency in citation formatting (some references use "et al." while others list all authors).

Response: Thank you for your comment. We have thoroughly reviewed the references and ensured consistency in formatting. For references with more than six authors, we have listed the first six authors followed by "et al." as per the guidelines.

**Review Comments to the Author (mentioned in the email)**

Reviewer #2: (No Response)

**Response:** Not required

Reviewer #3: Authors have addressed all the comments though few are clarified why they have mentioned and can't be modified further. Just one comment to author: Reflect the overall findings in conclusion of the abstract, so that your takeaway key message is heard by readers of policy makers of Bhutan.

**Response:** Thank you for your valuable feedback. We appreciate the suggestion to ensure our key findings are clearly conveyed to policymakers. In response, we have revised the conclusion in the abstract to better reflect the overall findings. The amended version now reads:

“Overall, with a score of 4.06 on a 5-point Likert scale, patient satisfaction in Bhutan is high. However, our findings highlight the need to address socio-demographic disparities in patient satisfaction. As the Bhutanese socio-demographic landscape evolves, satisfaction levels may decline. To enhance overall satisfaction, healthcare policymakers should focus on improving accessibility and convenience. Strategies such as establishing dynamic limits on free services, exploring private sector engagement in advanced healthcare service, and strengthening the healthcare workforce are essential for sustainable and quality healthcare service delivery.”

We hope this revision effectively conveys the key message to policymakers and stakeholders.

Reviewer #4: (No Response)

**Response:** Not required

Reviewer #5: Definitely this study will provide a baseline for many more studies that can be carried out in the future.

**Response:** Not required

Reviewer #7: (No Response)

**Response:** Not required

Reviewer #9: All comments of the reviewers have been addressed. Please also read this and cite in the discussion: <https://www.taylorfrancis.com/chapters/oa-edit/10.4324/9781003187462-17/negotiating-covid-19-bhutan-mary-grace-pelayo-ian-christopher-rocha-jigme-yoezer>

**Response:** Thank you for the suggested reference. We have carefully reviewed the article and appreciate its insights. Upon evaluation, we found that the key information relevant to our study has already been incorporated using the latest available references or original source. Therefore, we feel that adding this citation may not provide additional value to the manuscript. However, we sincerely appreciate the recommendation and hope this clarification addresses the reviewer’s suggestion.
