# Supplementary material for: Patient satisfaction in regional referral hospitals of Bhutan: Insights from a cross-sectional study
Source: PLoS One. 2025 Jun 2;20(6):e0312629. doi: 10.1371/journal.pone.0312629 (PMC12129210; doi:10.1371/journal.pone.0312629)
Supplement: Attachment — Submitted filename: Acceptance letter.docx [file pone.0312629.s005.docx]

Date: 23/4/2025

To

The Editor,

PLOS One

California, US

**Subject: Review Completion for Manuscript ID - PONE-D-24-44865R2**

Dear Editor,

Thank you for inviting me to review this manuscript again. I have reviewed the revisions and the authors' responses to my previous comments. At this stage, I have no further substantive feedback to add, as my earlier concerns have been adequately addressed.

If there are specific aspects the journal would like me to re-evaluate, please let me know. Otherwise, I defer to the editorial judgment on whether the manuscript is now suitable for publication.

Best regards,

Kinley Gyem
